# Supplementary material for: The Cannabinoid CB1 Receptor Inverse Agonist/Antagonist SR141716A Activates the Adenylate Cyclase/PKA Signaling Pathway Among Other Intracellular Emetic Signals to Evoke Vomiting in Least Shrews (Cryptotis parva)
Source: Int J Mol Sci. 2025 Oct 11;26(20):9884. doi: 10.3390/ijms26209884 (PMC12564499; doi:10.3390/ijms26209884)
Supplement: Supplementary file 1 [file ijms-26-09884-s001.zip › ijms-3866285-supplementary.pdf]

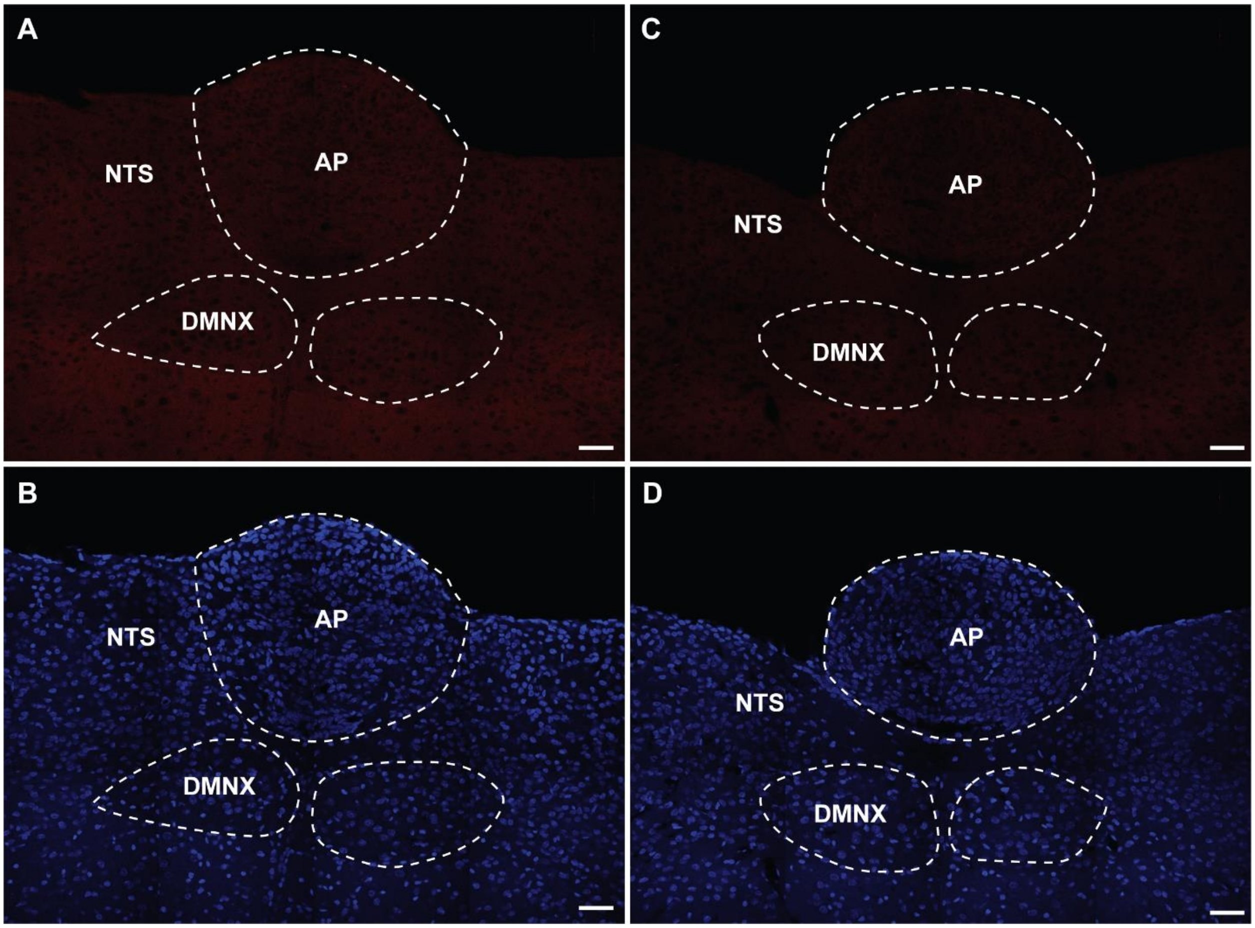

Figure S1. Negative controls for CB1 immunofluorescence in the least shrew brainstem (dorsal vagal complex). Representative coronal sections through the dorsal medulla showing the area postrema (AP), nucleus tractus solitarius (NTS), and dorsal motor nucleus of the vagus (DMNX); dashed lines delineate the ROIs used for quantification. A–B, host-matched rabbit IgG isotype control in place of the primary CB1 antibody (A, red detection channel; B, DAPI nuclear counterstain). C–D, secondary-only control with omission of the primary antibody (C, red detection channel; D, DAPI). All images were acquired with identical laser power, gain, and post-processing settings as the experimental CB1 images to document background levels. Minimal background signal is observed within AP, NTS, and DMNX under both control conditions. Scale bar: 50 μm.
